# Optimization of stereotactic radiotherapy (SRT) planning for brain metastases from lung cancer by Cyberknife system: reducing dose distribution inner ear

**DOI:** 10.7150/jca.92186

**Published:** 2024-01-01

**Authors:** Hua Zhang, Xuyao Yu, Yuwen Wang, Zhiyong Yuan, Yang Dong, Xiaoguang Wang

**Affiliations:** 1 Department of Lung Cancer, Tianjin Medical University Cancer Institute and Hospital, National Clinical Research Center for Cancer, Key Laboratory of Cancer Prevention and Therapy, Tianjin's Clinical Research Center for Cancer, Tianjin Lung Cancer Center, Tianjin, 300060, People's Republic of China.; 2 Department of Radiation Oncology, Tianjin Medical University Cancer Institute and Hospital, National Clinical Research Center for Cancer, Tianjin's Clinical Research Center for Cancer, Key Laboratory of Cancer Prevention and Therapy, Tianjin, 300060, People's Republic of China.; 3 Department of Radiotherapy, Tianjin Cancer Hospital Airport Hospital, Tianjin, 300308, People's Republic of China.; 4 Department of Brain Oncology, Tianjin Medical University Cancer Institute and Hospital, Tianjin, 300060, People's Republic of China.

**Keywords:** CyberKnife, Stereotactic radiotherapy, Brain metastases, Inner ear

## Abstract

**Objective:** Through retrospective statistical analysis of radiation distribution in inner ear avoidance for brain metastases from lung cancer by the CyberKnife (CK) system, it can provide a reference for stereotactic radiotherapy (SRT) planning and treatment optimization.

**Methods:** Computed tomography/magnetic resonance imaging data of 44 patients with one brain metastases lesion from lung cancer were used to re-plan and analyze, who had been treated by CK system from April 2021 to April 2022. The prescribed doses of 14-30 Gy in 1-3 fractions was simultaneously delivered to the metastatic lesions. The SRT plans for the same patients were replaned under with and without inner ear avoidance setting. The plan parameters and dose distribution differences were compared between plans.

**Results:** All plans met the dose restrictions. There were no significant differences in the coverage (*Coverage*), conformity index (*CI*), mean dose (D_mean_), the maximum dose (D_max_) and minimum dose (D_min_) of planning target volume (PTV). With inner ear avoidance setting, the D_max_ and D_mean_ of inner ear area decreased by 13.76% and 12.15% (*p*<0.01), respectively. The total number of machine nodes and monitor units (MU) increased by 4.63% and 1.06%.

**Conclusions:** During the SRT plan designing for brain metastases from lung cancer, the dose distribution in inner ear area could be reduced by avoidance setting, and the patient's hearing would be well protected.

## Introduction

Brain metastases is the most common distant metastasis sites of lung cancer. The whole brain radiotherapy (WBRT) and stereotactic radiotherapy (SRT) are currently common clinical treatments. Previous studies have shown that the survival time of patients receiving simple SRT was comparable to that of WBRT+SRT 1. Cyberknife (CK) is a common SRT system used for stereotactic radiotherapy (SRT) of brain metastases. The orthogonal X-ray imaging device of CK can be obtained in real time image of the patient's skull during treatment, which can ensure treatment accuracy and achieve high-dose irradiation of the target area. However, the high prescription dose of SRT means that organs at risk (OARs) may receive higher radiation doses 2. In addition, as the survival period of patients increases, new brain metastases in patients with lung cancer brain metastasis after receiving SRT. Most lung cancer brain metastasis patients could need for another SRT or whole brain radiotherapy. Therefore, more attention should be paid to the dose of OARs in the patient's SRT plans.

Clinical studies have shown that the degree of hearing loss in patients is closely related to radiation dose received in inner ear 3. The threshold cochlear dose for hearing loss with chemotherapy and radiotherapy combination was predicted to be 10 Gy 4. There is currently no effective way to alleviate or treat hearing impairment of patients. Therefore, it is necessary to find a safe and effective treatment plan optimization and irradiation method to achieve radiation protection for inner ear area during the SRT treatment of brain metastases from lung cancer.

This study retrospectively analyzed the CK treatment plans of 44 brain metastases patients from lung cancer. The dose distribution differences in inner ear area were compared between with and without inner ear avoidance setting plans, providing clinical reference for brain metastases patients CK SRT planning.

## Materials and Methods

### Clinical data

This study retrospectively evaluated the data of 44 brain metastases patients from lung cancer, the lesions with a range of 3 cm from ear structure (cochlea, vestibule, internal auditory canal, tympanic cavity, and bony eustachian tube). They received SRT using CK from April 2021 to April 2022, in Tianjin Medical University Cancer Institute and Hospital. Table 1 shows the patient characteristic, including 26 males and 18 females.

The inclusion criteria were:

① Histologically and/or radiologically proven non-small cell lung cancer,

② No other malignancies diagnosed within 5 years,

③ Absence of nodal and metastatic disease,

④ Brain metastasis confirmed by magnetic resonance imaging (MRI).

The exclusion criteria were:

① Received systemic chemotherapy,

② Received whole brain radiation therapy,

③ Received surgery for brain metastasis site.

This study has been approved by the Ethics Committee of the Cancer Hospital of Tianjin Medical University, and the patient's informed consent has been obtained and signed.

### SRT Plan Design

All patients were in supine position wearing a thermoplastic mask (CIVCO, Orange City IA, USA) before computed tomography (CT) simulation. Overall skull CT scan for the patients was performed by GE Discovery RT590, with a slice thickness of 1.25 mm. T1-weighted MRI axial sequences were acquired using a Siemens 1.5T scanner. The CT and MRI datasets of patients were imported registered in the Precision 1.1.1.1 planning system (Accuray Inc., Sunnyvale, CA, USA) for the gross tumor volume (GTV) and OARs delineation. Planning target volumes (PTV) were obtained by expanding 1.5 mm of GTV in three dimensions. The OARs included: the eyeball, lens, optic nerve, optic chiasm, brainstem, and inner ear (cochlea, vestibule, inner ear, tympanic cavity, and bony eustachian tube). All structures for SRT planning were reviewed and approved by two independent experienced radiation oncologists and a neurosurgeon.

Two different treatment plans were designed for every patient, with and without inner ear avoidance setting. The plans were designed with raytracing (RT) algorithm and 6D Skull tracking method. The same collimator size and prescription isodose line (65-70%) were adopted in both plans for the same patients. All the planes needed prescription dose coverage greater than 95% of PTV volume. And the PTV received 1400-3000 cGy (median 2200 cGy) in 1-3 fractions (median 2 fractions). The same constraint conditions were applied to OARs, and the single dose limit of segmentation included: the maximum dose (D_max_) of optic pathway (including optic nerve and optic chiasma) < 10 Gy and volumes receiving 8 Gy (V_8Gy_) < 0.2 cc, D_max_ of brainstem was < 15 Gy and volumes receiving 10 Gy (V_10Gy_) < 0.5 cc, hippocampal D_max_ ≤ 17 Gy and the mean dose (D_mean_) of inner ear ≤ 15 Gy. The radiotherapy path in SRT plans was set to prohibit transmission through patients' lens, so that the lens can be well protected (D_max_<1Gy).

### Evaluation of treatment plans

Design results of SRT plans with and without inner ear avoidance setting, the coverage (*Coverage*) and conformity index (*CI*) of PTV were evaluated and compared. Statistics and analysis of dose distribution differences in OARs included: hippocampal, inner ear, optic pathway, brainstem and lens. The total beam node and total monitor units (MU) were compared.

### Statistical methods

The plan parameters conformed to normal distribution, and the results were expressed in the form of mean ± standard deviation (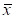
±s). Student t test was used to compare the pairwise pairwise between the planning parameters, and *p* -value < 0.05 was considered as statistically significant.

## Results

### Planning parameter Differences

From the results in Table 2, it can be seen that the *CI* and *Coverage* obtained by two different plans are similar, and there is no statistical difference (*p* > 0.05). This indicates that adding inner ear avoidance setting does not affect the dose distribution of the PTV, prescription dose covering the PTV in all plans can meet the clinical requirements.

The total number of machine nodes and total MU in limit setting plans were higher than the other plans, with an average increase of 4.63% and 1.06%, respectively (*p* < 0.05). This means that inner ear avoidance setting may limit the passage of rays through the inner ear area. In order to ensure that the PTV receives prescribed doses, SRT plan design could choose more machine nodes to transfer radiation dose. At the same time, it will increase total MU in the plans. The total number of machine nodes and total MU increase indicates longer treatment time for patients.

### Dose distribution differences in OARs

Table 2 is the dose distribution results of OARs in two different plans. It can be seen that the SRT plans with inner ear avoidance setting can reduce D_max_ and D_mean_ of inner ear 13.76% and 12.15%, respectively (*p* < 0.01). It means that SRT plans with inner ear avoidance setting can protect the patient's hearing system, reduce the risk of radiation hearing damage. Although the decrease in dose distribution of inner ear area possibly result in a slightly elevated dose distribution around hippocampus and brainstem. That is still within an acceptable range. Therefore the inner ear avoidance setting of SRT plans for brain metastases near the ear structure can improve the quality of life of patients after receiving radiotherapy.

## Discussion

SRT is a three-dimensional localization irradiation for patients' lesions through stereotactic technology, and has become one of the commonly used treatment methods for brain metastases from lung cancer in clinical practice 5. Because of the short time and high dose irradiation in SRT plan, the OARs near the target could be exposed to higher radiation dose. When the eustachian tube and middle ear mucosa are exposed to high radiation, those structures will develop inflammatory edema. That can cause eustachian tube obstruction, resulting in increased negative pressure in the middle ear. It will eventually induce radiation otitis media in patients after SRT. Some studies have shown that about 12.6% of nasopharyngeal carcinoma patients will develop radiation otitis media after radiotherapy 6. Hsin CH et al. showed that compared with traditional radiotherapy, intensity modulated radiotherapy (IMRT) is more likely to cause radiation damage to important structures such as the middle ear, eustachian tube and palatine veli levator muscle of patients, resulting in eustachian tube dysfunction, negative pressure formation in the tympanic chamber and secretory otitis media 7-8. The study of Parham K showed that the degree of apoptosis of inner ear cells was closely related to the radiation dose and duration 9. Jereczek-Fossa B A et al. had shown that up to 50% of patients with head and neck tumors would develop sensorineural deafness after 1 year of radiotherapy, and permanent hearing loss would occur in severe cases 10. Therefore, in the design and implementation of SRT treatment plan for intracranial tumor patients, attention should be paid to the protection of ear structure.

The CyberKnife (CK) system uses a 6 degrees-of-freedom robotic arm to drive a 6MV accelerator for radiotherapy 11][12. Therefore, when the CK system is used to design SRT plan for brain metastatic tumor near the ear structure, the high irradiation dose of the target area can be achieved while the irradiation dose of the normal brain tissue around the target area can be minimized by selecting different nodes of the system and optimizing the number of machines on the nodes. Through the design and analysis of different CK SRT plans of 44 brain metastases from lung cancer, we found that adding the ear structure dose limits in the SRT planning could effectively reduce dose distribution in this area, while ensuring the clinical treatment effect. In terms of treatment plan parameter evaluation, CK SRT plans with and without dose limits of inner ear structure also had good *CI* and *Coverage* of the PTV, and there was no significant difference between them. This means that adding dose limits did not cause differences in dose distribution of PTV. The total number of beam nodes and MUs were increased in the SRT plans with dose limits of inner ear structure. It suggested that in order to protect the inner ear area, CK system distributed the prescribed dose to more treatment nodes and the implementation of the plan needed more time.

In summary, during SRT planning for brain metastases near the inner ear structure, setting the dose limits for inner ear area can effectively reduce the radiation damage for the hearing system of patients, while ensuring the dose distribution in the target area and the therapeutic effect. However, the study of clinical treatment effect before and after the optimization of SRT plan still needs to further track more clinical cases and follow-up records for discussion, so as to establish a reliable clinical database and provide a more beneficial reference for the clinical SRT treatment of patients with head and neck tumors.

## Figures and Tables

**Table 1:** A total of 44 patients with brain metastases patients from lung cancer, the lesions with a range of 3 cm from the hippocampus or ear structure.

Characteristic	Cases
Age	
<50 year	19 (43.18%)
≥50 year	25 (56.82%)
Tumor diameter	
<3 cm	38 (86.36%)
>3 cm	6 (13.64%)
Tumor volume	
<100 ml	37 (84.09%)
>100 ml	7 (15.91%)

**Table 2:** Evaluation parameters of 44 brain metastases from lung cancer plans with and without inner ear avoidance setting

	Plans without limit setting Mean (range)	Plans with limit setting Mean (range)	*R* Mean (range)	*t*	*P*
*CI*	1.13 (1.08 ~ 1.26)	1.14 (1.08 ~ 1.27)	*	-1.74	0.37
*Coverage*	95.39 (95.32~96.51)	95.37 (95.32~96.49)	*	4.51	0.55
Beam node	104 (100 ~ 131)	112 (104 ~ 145)	-7.69% (-4.00% ~ -10.69%)	2.36	0.04
Monitor units (MU)	17418.29 (9375.57 ~ 20380.79)	18835.14 (9742.16 ~ 22413.96)	-8.13% (-3.91% ~ -9.96%)	-5.19	0.04

Note: "*" indicates that there is no significant difference between with and without inner ear avoidance setting plans.

**Table 3:** Dose distribution of OARs in 44 brain metastases from lung cancer SRT plans with and without inner ear avoidance setting (%, 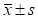)

		Plans without limit setting	Plans with limit setting	*R*	*t*	*P*
Inner ear	D_max_	52.41±4.83	45.20±5.93	13.76±2.30	4.57	<0.01
	D_mean_	28.19±9.45	24.82±6.27	12.15±2.91	3.66	<0.01
Hippocampal	D_max_	29.04±5.27	29.19±4.09	*	6.12	0.81
	D_mean_	16.12±6.01	16.69±6.44	*	1.17	1.84
Lens	D_max_	1.22±3.71	1.49±3.02	*	-5.12	0.52
Brainstem	D_max_	22.73±5.18	22.95±4.96	*	-4.24	2.26
	D_mean_	15.02±4.47	15.23±4.01	*	1.74	1.7
Optic pathway	D_max_	15.34±5.57	15.17±4.79	*	3.29	1.91
	D_mean_	7.91±3.08	7.76±5.27	*	4.47	0.36

Note: Dose data in the table represents the percentage of the highest dose in SRT plans, the dose parameters of inner ear is the unilateral area close to the tumor, "*" indicates that there is no significant difference between with and without inner ear avoidance setting plans.

## Abbreviations

SRT: stereotactic radiotherapy
CK: CyberKnife
OARs: organs at risk
CI: conformal index
PTV: planned target volume
GTV: gross target area
D_mean_: mean dose
D_max_: maximum dose
WBRT: whole brain radiotherapy
MRI: magnetic resonance imaging
CT: computed tomography
V_10Gy_: volumes receiving 10 Gy
V_8Gy_: volumes receiving 8 Gy
MU: monitor units
